# Adaptation and validation for use in Brazil of the Confusion, Hubbub, and Order Scale (CHAOS)

**DOI:** 10.1186/s41155-024-00310-5

**Published:** 2024-07-15

**Authors:** Marilia Ignácio de Espindola, Maria Laura Nogueira Pires, Renatha El Rafihi-Ferreira, Ana Regina Noto, Sabine Pompéia

**Affiliations:** 1 Departamento de Psicobiologia, Universidade Federal de São Paulo (UNIFESP), Campus São Paulo, São Paulo, SP Brazil; 2 Núcleo de Pesquisa Em Saúde E Uso de Substância (NEPSIS), São Paulo, SP Brazil; 3 Faculdade de Ciências E Letras, Universidade Estadual Paulista (UNESP), Campus de Assis, São Paulo, SP Brazil; 4 Departamento de Psicologia Clínica, Universidade de São Paulo (USP), Campus São Paulo, São Paulo, SP Brazil

**Keywords:** CHAOS, Scale validation, Adolescent, Problem behavior, Socioeconomic factors, Stress, Psychological

## Abstract

**Background:**

The Confusion, Hubbub, and Order Scale (CHAOS in English Version) was originally developed in the USA by Matheny et al (Bringing order out of chaos: psychometric characteristics of the confusion, hubbub, and order scale. Journal of Applied Developmental Psychology 16(3):429–444, 1995) to measure chaos in the family environment, characterized by confusion, lack of routine, and organization.

**Objective:**

To present evidence of content validity, internal structure validity, and validity based on relationships with external measures of an adapted version of the CHAOS into Brasilian Portuguese with adolescents sample in São Paulo - Brasil.

**Method:**

Study 1 involved the translation/back-translation and adaptation of the scale into Brazilian Portuguese [here named “Escala de Confusão, Alvoroço e Ordem no Sistema familiar” (CAOS)], assessed by 5 judges. In Study 2, we conducted an exploratory factor analyses (EFA) to determine the scale’s factor structure (*N* = 180 adults). In Study 3, we carried out confirmatory factor analyses (CFA) to confirm the internal validity of the scale, along with complete structural equation modeling to explore convergent validity in another sample (*N* = 239 adolescents).

**Results:**

The CAOS scale displayed content validity, and the EFA and CFA showed a unifactorial structure (with some scale adjustments) with an acceptable fit. The family chaos latent factor was associated with externalizing symptoms and perceived stress in adolescents.

**Conclusion:**

Overall, the Brazilian version of the scale presented evidence of construct, internal, and concurrent validity that indicate its usefulness in Brazil.

**Supplementary Information:**

The online version contains supplementary material available at 10.1186/s41155-024-00310-5.

## Introduction

During childhood and adolescence, the family microenvironment is of vital importance, as it is at home that the main interactions between developing individuals and their caregivers occur (Matheny et al., 1995; Wachs, 1989). Some factors in this environment can negatively affect healthy development, such as the level of chaos in the family environment. In Bronfenbrenner’s bioecological model, the term “chaotic systems” is also used, indicated by frenetic and unpredictable daily activities and, lack of routine and structure (Bronfenbrenner & Evans, 2000). A chaotic microenvironment has a great propensity to disrupt proximal processes, an important concept in bioecological theory that is defined, broadly speaking, as peoples’ experiences including their activities, roles, and personal relationships in their immediate environment (Bronfenbrenner & Evans, 2000; Bronfenbrenner & Morris, 1998; Wachs & Evans, 2010). Chaotic environments can also generate a calibration in the stress response of young people so that they can better deal with unpredictability (Ellis & Del Giudice, 2019).

To measure chaos in a family environment, Matheny et al. (1995) developed the Confusion, Hubbub, and Order Scale (CHAOS) based on the characterization of family chaos as this microenvironment’s potentially stressful, nonspecific background physical factors, such as noise, the intense flow of people entering and leaving the house (Wachs, 1989), as well as disorganization and lack of routine (Tucker et al., 2017; Wachs & Evans, 2010). This scale contains 15 items (statements) evaluated dichotomously (true vs. false) that measure the respondents’ perception of their family environment from which a single score is obtained. However, note that there are no gold-standard measures of this broad concept of chaotic family environment, which has made it impossible as yet to ascertain the criterion validity of the CHAOS. Nonetheless, this scale has been translated into various languages worldwide and is widely used in the international literature (Marsh et al., 2020). In part, this is because researchers have found that high scores on the CHAOS and its short versions are associated with various negative outcomes during childhood and adolescence, as predicted theoretically from the impact of growing up in a confusing microenvironment (Bronfenbrenner & Evans, 2000; Bronfenbrenner & Morris, 1998; Matheny et al., 1995; Wachs & Evans, 2010). These outcomes include cognitive deficits (Andrews, et al., 2021a, 2021b), impaired academic performance (Hanscombe et al., 2011; Marsh et al., 2020), behavioral problems (Vilsaint et al., 2013; Wang et al., 2012; Wilhoit et al., 2021), and risk-taking such as excessive drug consumption (Chatterjee et al., 2015; Delker et al., 2020). The ability of the total scale’s score to associate with and/or predict these negative outcomes therefore made it a valuable instrument for studying the impact of chaotic family environments throughout development.

Another aspect of the CHAOS that makes it popular is its test-retest stability (Matheny et al., 1995) and reliability, with the scale demonstrating high internal consistency (Matheny et al. (1995), a measure of how closely related the items within a scale are to one another in measuring the same construct or concept. This psychometric property is usually assessed using Cronbach’s alpha index. For example, in the article in which the CHAOS scale was proposed, this index was α = 0.79 (Matheny et al., 1995). However, this metric is currently considered inadequate for this purpose, as proposed by Sijtsma (2009) and the American Educational Research Association (AERA), American Psychological Association, and National Council on Measurement in Education (AERA et al. 2014). One of the reasons for this is that this metric assumes that the tested scale measures a single construct and that all scale items have the same weight in determining the overall score (the tau equivalence principle) (Cronbach, 1951), neither of which have been confirmed. After all, scale items that share variance can reflect the existence of an underlying construct (latent factor or variable), but more than one such factor can be found in a given scale (Brown, 2015; Damásio, 2012).

To evaluate the internal consistency and factor structure of a scale it is necessary to conduct: (1) an exploratory factor analysis (EFA), a set of multivariate techniques that aim to find (with exploratory methodology) the number of latent factors present in a scale, (Brown, 2015; Damásio, 2012). Also, from an EFA it is possible to determine the internal consistency of a scale using indices such as the Composite Reliability index (Raykov, 1997); and (2) a confirmatory factor analysis (CFA), a type of structural equation modeling in which the factor structure is defined a priori, to confirm results of EFAs and/or validate whether a specific factor structure aligns with the theoretical framework under investigation (Brown, 2015). In both EFAs and CFAs, it is assumed that a scale assesses a single (one-factor) construct when all items share variance. However, if a scale presents items that reflect constructs that are different at the latent level, this indicates that the answers to a scale reflect more than one factor or construct (which may or may not correlate with each other).

Although Matheny et al. (1995) mention that confusion in the family environment includes “ambient noise, crowding, and environmental traffic patterns”, they did not describe how this mapped onto the items that enquired about each of these aspects, nor analyzed the results regarding these separate issues, having used a score that adds points considering all items. Therefore, we found that there was no basis for considering that the original authors of the CHAOS intended these three aspects of environmental confusion to be treated as different constructs of the scale. Additionally, the fact that Matheny et al. (1995) used Cronbach’s alpha to determine the internal consistency of the CHAOS scale, a metric that necessarily considers intercorrelations among all its items, suggests that they conceived it as a single-factor scale, but this was not made explicit in any publication and there is not literature on the proposal of subdivisions of family chaotic environments in theoretical terms to the best of our knowledge.

Notwithstanding, the existence of more than one factor or construct in the CHAOS scale could be found psychometrically using EFAs and CFAs, although there are only a couple of studies that explored the internal structural validity of the CHAOS scale using alternative metrics to Cronbach’s alpha. One of these was a dissertation on a North American sample (Shervey, 2013) in which various sets of models with one, two, and three factors were tested, with no sound theoretical bases. To this end Shervey (2013) used CFA, but none of the models had acceptable fit indices. Sánchez-Mondragón and Flores Herrera (2019), in a Mexican validation, sought to establish a configurable structure of the scale with three factors based on Shervey’s (2013) results, despite the lack of theory to establish these factors and the inadequacy of the models in her study. Sánchez-Mondragón and Flores Herrera (2019) did obtain acceptable adjustment indices, which should have indicated the existence of three separable constructs measured by the scale, but this model only included nine of the 15 items. Also, instead of true/false responses in the original CHAOS scale, a 4-point Likert scale was used, so this does not supply information on the factor structure of the CHAOS *as it was originally conceptualized*. We therefore stress that in neither of these two studies, a theoretical justification for the existence of more than one latent factor was provided, nor why specific items of the scale used to measure each proposed factor. In sum, there is not enough data on the internal consistency of the CHAOS (consistent pattern of inter-associations among items, which can have different weights) using more up-to-date psychometric methods (Raykov, 1997), nor reliable information on its factorial structure.

Therefore, it remains necessary to provide evidence of the validity of the internal/factor structure of the CHAOS, that is, to determine the degree to which responses to its items indicate the dimensionality of the construct under evaluation. This can be done using EFA and CFA (AERA et al. 2014; Brown, 2015), which we undertook here. Another way to determine the validity of a scale, which was also explored, is to examine the extent to which latent scores relate to other measures or constructs as this can provide evidence of convergent validity (AERA et al. 2014). In the case of the CHAOS scale scores, its total (raw) score is positively related to some variables that will be explored here, namely: higher number of internalizing and externalizing behavior problems (Wang et al., 2012; Wilhoit et al., 2021), and higher levels of stress (Brown et al., 2019; Doom et al., 2018; Ellis & Del Giudice, 2019) in children and adolescents. Moreover, a lower family socioeconomic status (SES) is associated with higher family chaos raw scores, possibly due to factors such as the accumulation of physical and social stressors, the effects of poverty, and lack of access to health assistance (Brown et al., 2019; Doom et al., 2018; Evans et al., 2010; Marsh et al., 2020; Philbrook et al., 2020). Therefore, it can be assumed that one or more latent variables that represent chaos in the family environment are likely associated with all these factors. However, it should be noted that lower SES is also associated with more internalizing, externalizing, and stress symptoms (Korous et al., 2018; Marsh et al., 2020) which are, in turn, also related to family chaos, as mentioned above. Therefore, in countries such as Brazil, where there is high social inequality, it is important to consider the effects of family chaos on these behavioral outcomes controlling in some way family differences in SES.

Given the lack of investigations into the reliability (internal consistency), factorial structure, and validity of the CHAOS scale in the international literature using current psychometric techniques, and the lack of instruments in Brazil that can be used to assess chaos in the family environment, our aim was to provide such evidence in a cultural adaptation of this scale to Brazilian Portuguese, which we named the “Confusão, Alvoroço e Ordem no Sistema familiar” (CAOS) scale. We strived to do so keeping the scale as close as possible to its original version with respect to the meaning of the items in Portuguese, including reverse scoring of the same items in the version in English so that results could be comparable to those of other studies that employed the same scale. Nonetheless, we anticipated that it would possibly be necessary to remove some items that did not present adequate psychometric properties. Matheny et al. (1995), for instance, reported that the raw scores of item 15 were only marginally correlated with the total sum score of the scale, while item 13 also had lower correlations with the total score compared to the remaining items, suggesting they contributed little to the underlying construct.

To achieve these objectives, three studies were carried out:

- *Study I*: assessed evidence of content validity based on the translation and cultural adaptation of the Confusion, Hubbub, and Order Scale (CHAOS) from the original version in English into Brazilian Portuguese.
- *Study II*: assessed evidence of validity based on internal structure with exploratory factor analysis (EFA) and determined the factorial structure of the version of the scale in Portuguese in a sample of mothers of children.
- *Study III*: evaluated additional evidence of validity based on internal structure using confirmatory factor analysis (CFA) with the aim of confirming the factorial structure identified in the exploratory factor analysis (EFA) in a different sample to that used in Study II, including adolescents. Additionally, we assessed convergent validity in this sample by determining the relationship between latent scores on the CAOS scale and other latent scores, including (a) socioeconomic status (SES), measured by the average years of parental education; (b) internalizing and (c) externalizing behaviors in adolescents assessed by the Child Behavioral Checklist (CBCL: Achenbach, 1991; Bordin et al., 2013) answered by parents; and (d) self-reported stress by adolescents using the four-item Perceived Stress Scale (Faro, 2015; Warttig et al., 2013).

Given that three studies were conducted, the Methods, Results/Discussion of each one will be presented separately below in a more specific manner, followed by an overall General Discussion regarding all findings together and their application towards considering the appropriateness of the Brazilian version (CAOS) of the CHAOS scale based on the international literature outlined in the Introduction.

## Study I: translation and adaptation of the CHAOS scale for use in Brazil

### Method

#### Participants

Five judges were involved in the entire translation and re-translation process plus authors MLPN and RER, all of whom were highly proficient in both Portuguese and English.

#### Procedure

To the best of our knowledge, the CHAOS scale (Matheny et al., 1995) has no copyrights and does not require authorization to be translated, adapted, and validated for other cultures. Nevertheless, we tried to contact the original authors to request approval but had no answer until the publication of this work.

The translation and cultural adaptation procedure aimed to verify content validity. The process of assessing semantic equivalence and scale adequacy involved four stages (Reichenheim & Moraes, 2007) carried out by experts: (1) translation; (2) back-translation; (3) the assessment of equivalence between the translated and origin items; and (4) the preparation of the final version of the Brazilian version of the scale based on the selection of the most adequate back-translations/translations obtained in stage 3.

Stage 1 consisted of two independent translations of the original instrument in English into Portuguese. The first version (V1) was translated by a medical doctor fluent in English and Portuguese, and the second (V2), by a bilingual professional with experience in the area.

In stage 2, versions V1 and V2 were both back-translated into English by highly proficient bilingual professionals with a good understanding of Brazilian culture and whose native language was English (back-translation 1) and by a professional translator (back-translation 2). The back-translations were also done independently with no access to the original scale.

In stage 3, the aim was to assess two aspects: the semantic equivalence and overall equivalence of the translations with respect of the original version. This stage was conducted by a bilingual professional with a degree in linguistics who evaluated the equivalence between the original version and each of the back-translations, considering both referential meanings (R = corresponding to a literal equivalence between item pairs in both languages) using a visual analog scale ranging from 0 to 100%, and general meaning (G = corresponding to a broader agreement between these pairs) judged at four levels—unchanged, slightly changed, considerably changed, or extremely changed. To this end, the two back-translated versions of each item were arranged randomly beside the original items so that it was not possible to identify which of the two back-translations originated from each of the two experts. To reduce the possibility of biased choices, note that this expert was also not involved in the translation and back translations, nor the study itself beyond this task. Based on this expert’s responses, stage 4 involved selecting the back-translated items that were most faithful to those of the original CHAOS scale and also the translated version that was the most clear and unambiguous in Portuguese. This was undertaken by authors MLPN and RER who also were proficient in both languages and had a deep understanding of the concepts associated with family chaos. Instead of focusing on specific terms in Portuguese and English, these authors relied on the linguists’ professional expertise to select the best-translated version as a whole.

### Results and discussion

The general referential meaning was found to be unchanged in stage 3 in seven of the 15 items (items 2, 6, and 7, 8, 12, 13, and 14) which obtained referential scores of 95–100% (general meaning unchanged) in both back-translated versions, whereas another five items had scores of 90% (slightly changed general meaning) versus 100% (items 3, 45, 9 and 10). In these cases, the translated versions that either received higher scores or were easier to understand by authors MLPN and RER in stage 4 were selected for inclusion in the Portuguese version of the instrument. Only two items (11 and 15) had lower scores (60%; considerably changed meaning) in one of the two back-translations, so the alternative versions (scores 100%) were selected in stage 4 to compose the Portuguese version. There were two exceptions in stage 4: (1) it was considered that versions 1 and 2 of item 5 of both the back-translations complemented each other, so the final version was adapted by amalgamating the two versions; and (2) both translated/back-translated versions of item 8 were deemed unchanged and with good referential meaning, but one of them corresponded to item 6 so the alternative version was selected to be included in the final version. Using these results, the final version of the instrument called the CAOS scale in Portuguese, was obtained, which can be found in Additional file 1: Table 2A.

## Study II: determination of the factorial structure of the CAOS scale with exploratory factor analysis (EFA) in a sample of mothers

This study involved carrying out EFA to explore the factorial structure of the translated version (CAOS) of the CHAOS scale because no previous studies with adequate psychometric methodology and grounded on theory were found on the factorial structure of the scale (see Damásio, 2012).

### Participants

A convenience sample of 180 literate adult Brazilian mothers of children aged between 6 months and 6 years from the city of Assis in Brazil.

### Procedure

The sample was drawn from the one in a study approved by the ethics committee of the Universidade Estadual Paulista Júlio de Mesquita Filho (Campus de Assis) (CAAE: 06959612.3.0000.5401), which included other measures that will not be addressed here. Only literate adult mothers (aged 18 years or older) of preschool children aged between 6 months and 6 years of age who were part of this study were eligible. All participants signed an informed consent form. They were invited to complete the translated final version of the CAOS scale (Additional file 1: Table 1A). In addition, they reported their age in years, and their children’s age and filled in a socioeconomic status questionnaire of the *Associação Brasileira de Empresas de Pesquisa* (ABEP, 2018 - Brazilian Association of Research Companies).

### Instrument

CAOS Scale [adapted from the CHAOS by Matheny et al. (1995) in the study I; see Additional file 1: Table 2A). The scale has 15 statements that are evaluated by the respondent as true (score 0) or false (score 1). In contrast to the traditional scoring method (raw scores: the gross sum of scores after inverting the values of items 1, 2, 4, 7, 12, 14, and 15 so that higher total scores indicate greater chaos) (Matheny et al., 1995), in the present case the scores of each item were used (zero or one) in the EFA, inverting the scores for the abovementioned items (higher scores indicated greater chaos).

### Statistical analysis

#### Exploratory factor analysis

The software used was FACTOR version 12.1.2 (Lorenzo-Seva & Ferrando, 2006). The analysis was implemented using a polychoric correlation matrix and the robust diagonally weighted least squares (RDWLS) extraction method (Asparouhov & Muthén, 2010; Gana & Broc, 2019), which is suitable for indicators with dichotomous responses.

The Hull method by Comparative Fit Index (Hull-CFI) was the technique used for factorial retention (determination of the number of factors) (Ceulemans et al., 2011; Lorenzo-Seva et al., 2011) because it aims to find a model with the best balance between fit and number of parameters. This method combines the use of traditional analyses such as factor retention, scree plot, fit (through CFI), degrees of freedom, and the estimation method across a range of factor solutions. The Robust Promin rotation method was chosen (Lorenzo-Seva & Ferrando, 2019), which adjusts the relative weights of diagonal elements in relation to off-diagonal elements, losing as little variance as possible in the process, and resulting in simpler and more stable solutions. The adequacy of the exploratory analysis was assessed using the fit indices root mean square error of approximation (RMSEA), the Comparative Fit Index (CFI), and the Tucker-Lewis Index (TLI). According to Hu and Bentler (1999) and Brown (2015), RMSEA values must be less than 0.08, with the upper confidence interval not reaching 0.10; CFI and TLI values must be above 0.90, preferably higher than 0.95.

The *H* index was used to assess factorial stability (Hancock & Mueller, 2001), which varies from 0 to 1, and determines how well the set of items represents a factor (Ferrando & Lorenzo-Seva, 2018). H values higher than 0.80 suggest that each latent variable is well-defined, while lower *H* values suggest poorly defined latent variables.

Explained variance, expressed as a percentage, signifies the degree to which factors contribute to the variance of a specific item. A greater proportion of explained variance suggests that the extracted factors more effectively capture the variability of the item (Tavakol & Wetzel, 2020). The factor loadings are the correlation of the item with the latent factor (the higher the value, the higher the correlation).

The Measure of Sampling Adequacy (MSA) index was also used per scale item, whose values below 0.50 suggest that the item does not measure the same domain as the others within a given factor and, therefore, should be removed (Lorenzo-Seva & Ferrando, 2021). As a measure of internal consistency, the Composite Reliability index (Raykov, 1997) was calculated using the composite reliability calculator on the website The Statistical Mind (Colwell, 2016).

To analyze the interpretability of the item correlation matrix, the Bartlett and Kaiser–Meyer–Olkin (KMO) sphericity test was used (Hutcheson & Sofroniou, 1999).

As per the literature in general, because we used several fit indices (Hu & Bentler, 1998) and metrics, we considered factor solutions as acceptable if the values of most indices metrics were adequate. For example, the p values of *χ*^2^ are highly sensitive to sample size, therefore indices with *p* < 0.05 are acceptable as long as most of the other indices have adequate values.

### Results and discussion

The database and analysis scripts can be found on the Open Science Framework (OSF) 305 platform (https://osf.io/nrp6z/?view_only=7a7c11fa25ca44fd96c56e98690fa852). The mothers’ ages ranged from 18 to 30 years (mean = 33.11; SD = 6.25). They were classified in terms of socioeconomic status according to ABEP (2019) as 80 participants from classes A, 49 from classes B and C, and 51 from classes D and E. The mean total raw score of the CAOS scale was 4.27 (SD = 3.05).

### Factor extraction

The Hull-CFI method (Lorenzo-Seva et al., 2011) indicated the extraction of one factor as appropriate (CFI = 0.91; degree of freedom = 90, scree test value = 21.100). The factor loadings of the EFA single-factor model can be seen in Table 1. The replicability estimate of the *H*-index factor score (Ferrando & Lorenzo-Seva, 2018) was 0.824, the explained variance (the proportion of variance in each factor) was 0.23 and the composite reliability was 0.804). These metrics assess the validity of the internal structure of the one-factor solution for the scale and show it to be adequate.

**Table 1:** Factor loadings per item of the CAOS scale in the exploratory factor analysis (Study II; *N* = 180)

**Item**	**Factor loading**
1	0.529
2	0.376
3	0.365
4	0.174
5	0.388
6	0.356
7	0.470
8	0.693
9	0.302
10	0.355
11	0.387
12	0.595
13	− 0.016
14	0.757
15	0.114

For single-factor fit indices and other adequacy measures, see text. Factor loadings below 0.30 are regarded as inadequate (in grey)

### Factor loadings

To analyze the interpretability of the item correlation matrix in the single factor model (see Additional file 1: Table 3A), the Bartlett (521.4, df = 105, *p* < 0.001) and KMO (0.75) sphericity tests also suggested good adequacy of the matrix in respect of all items (Hutcheson & Sofroniou, 1999). The scale items also presented acceptable factor loadings (≥ 0.3: Field, 2020; Howard, 2016) with the exception of items 4, 13, and 15, which presented factor loadings lower than 0.30. Items 13 and 15 also had the lowest correlations with the sum score in the original paper that proposed the CHAOS scale (Matheny et al., 1995).

### Validity metrics

The fit indices of this one-factor model with all 15 items of the scale were acceptable (*χ*2 = 154.81, df = 90; *p* < 0.001; RMSEA = 0.06 (CI 0.04–0.06); CFI = 0.91; TLI = 0.89), but relatively poor fit. However, the factorial structure with all items on the scale reached an *H* value greater than 0.8, which suggests that the full set of 15 items explains the existence of a single factor well, indicating that this factor is replicable in future studies (Ferrando & Lorenzo-Seva, 2018). In addition, although the MSA index (Lorenzo-Seva & Ferrando, 2021) suggested the removal of items 3, 4, 13, and 15, which are those with the lowest factor loadings in this model (with the exception of item 3) (Howard, 2016), the composite reliability considering all 15 items was 0.80, which is considered acceptable (greater than 0.7: Marôco, 2021), even without removing the indicators with low factor loadings from the models. Thus, in order to try to maintain the original characteristic of the CAOS scale in relation to the original version in English, which contains all 15 items, it was decided to continue the analyses considering all the items of the scale for the next step, namely, the CFA to confirm the single-factor structure in another sample, as it is necessary to perform the CFA with different samples after performing EFA (Damásio, 2012).

## Study III: determination of the factorial structure of the CAOS scale with Confirmatory Factor Analysis (CFA) and Full Structural Equation Modeling (FSEM) in a sample of adolescents

This study involved carrying out a CFA with data from a sample of adolescents to confirm the EFA single-factor model solution obtained in study II in a sample of mothers, which must be undertaken in different samples. We believed that adolescents who were typically developing and in school years compatible with their age should have sufficient reading skills to understand and fill out the scale, especially because many of the mothers in study I had done so despite having low schooling/SES. We recruited adolescents from public and private schools to have a more representative sample in terms of variations in SES because we wanted to determine if SES was associated with family chaos. We began by testing a CFA including all the 15 scale items. Next, adjustments to the model and removal of some items were made to obtain a model with a better fit. The model with the best indices found in the CFA was then used to explore the validity of the internal structure, as well as to determine the interrelationship (convergent validity) of the latent score of family environment chaos with parental education (indicative of socioeconomic level), internalizing and externalizing symptoms, and perceived stress.

### Participants

A convenience sample of 239 typically developing adolescents from public and private schools in the city of São Paulo participated in this study. The exclusion criteria were (according to the reports of parents/caregivers described below): having been held back at school (repeated a year), being a student with special needs, or using medication regularly, in order to exclude participants with potential cognitive deficits and/or chronic diseases.

### Procedures

The study was part of a project approved by the ethics committee of the Universidade Federal de São Paulo (CAAE 56284216.7.0000.5505). Informed consent was obtained from the guardians/parents and informed assent from the adolescents. With the authorization of the schools, the young people and guardians were approached to explain the objectives of the study. The guardians who were interested in having their adolescents participate in the study, who also had to agree to do so, completed several demographic questionnaires to ascertain the eligibility of the adolescents. They then filled out other scales pertaining to the adolescents’ behavior and health. Among these was a scale (detailed below) that measure internalizing and externalizing behaviors reported by parents (Child Behavioral Checklist). The CAOS and the Perceived Stress Scale were answered by the adolescents (detailed below), individually in a separate environment from their classrooms at their own schools, along with other measures that will not be discussed here. The experimenter was always available to resolve any doubts the adolescents might have and to help them fill in the scales. All data was anonymized. All participants were reimbursed for transportation costs, received a Science Partner certificate, and the families were presented with a report on the findings that did not touch on any sensitive, confidential information provided by the youngsters to the experimenters. Referrals to health professionals were also provided when potential cognitive/physical health issues were identified.

### Instruments

#### Confusion, Hubbub and Order Scale (CHAOS)

The translated version (CAOS) of the original CHAOS scale developed by Matheny et al. (1995), available in Additional file 1: Table 2A, adapted for use in Brazil in study I and used in study II in mothers]. Here, it was filled in by adolescents. There was a change, however, in one item (item 8) in comparison to study II. A new bilingual expert involved in determining the suitability of the scale for adolescent respondents pointed out an ambiguity regarding the word “fuss” (defined by the Webster dictionary as “unnecessary activity or excitement often over something unimportant”). Therefore, the translated version of this item was changed accordingly. Additionally, for this population of adolescents, if their parents were separated, they were asked to consider the home in which they spent most of their time.

#### Parental education

The (self-reported) parents’ average years of education were used as an indicator of the SES of the adolescents' families (Farah, 2017; Korous et al., 2018).

#### The Child Behavior Checklist (CBCL)

Achenbach (1991); adapted for use in Brazil by Bordin et al. (2013): this scale contains 136 statements (items) about behavior that was filled in by parents regarding their offspring. Answers are provided using a 3-point scale (0 = not true; 1 = somewhat true or sometimes true; 2 = very true or often true) per item. Responses to various items are grouped to obtain scores of various types of symptoms. In the current study, only the raw scores for internalizing and externalizing symptoms were used. Internalizing behaviors include somatic complaints (11 items), depression/isolation (8 items), and depression/anxiety (13 items). Externalizing symptoms include rule-breaking behaviors (17 items), aggressive behaviors (18 items), social problems (11 items), thought problems (15 items), and problems related to attention (10 items).

#### The Perceived Stress Scale [PSS-4: Warttig et al. (2013); adapted for use in Brazil by Faro (2015)]

This reduced scale has four statements (items 2, 6, 7, and 14 from the 14-item version of the full PSS scale) about stress experienced in the last month and was completed by the adolescents. It uses a 5-point Likert scale with scores ranging from “never” (1 point) to “very often” (5 points). The total raw score is the sum of item scores after inverting the scores of two items (items 2 and 14 of the full scale) so that the higher the score, the greater the perceived stress.

### Statistical analyses

The database and scripts can be found on the OSF platform (https://osf.io/nrp6z/?view_only=7a7c11fa25ca44fd96c56e98690fa852). Missing data (5%) were imputed with the Mice package of Software R version 4.2.1 (Buuren & Groothuis-Oudshoorn, 2011). The logistic regression method was used for categorical variables and the cart method for continuous variables (Buuren & Groothuis-Oudshoorn, 2011).

#### Confirmatory factor analysis

The Mplus version 8.8 (Muthén & Muthén, 2017) software was used. Due to the dichotomous nature of the CAOS scale variables, the algorithm used to estimate the model was RDWLS (DiStefano and Morgan, 2014; Li, 2016). The model fit indices used for this analysis were *χ*^2^, RMSEA, CFI, TLI, and SRMR. A good fitting model should have *χ*^2^ *p* values lower than 0.05; the *χ*^2^/gl ratio must be < 5 or, preferably, < 3. For the other indices, the same cutoff values used for the EFA were used (Brown, 2015; Hu & Bentler, 1998). As per the literature in general, model solutions with adequate fit in most indices were considered acceptable. Furthermore, modification indexes (MI) were inspected and, if they were higher than 10, we included correlated errors between the items suggested by the statistical program in the models (Muthén & Muthén, 2017).

#### Complete Structural Equation Modeling (CSEM)

**Fig. 1:**
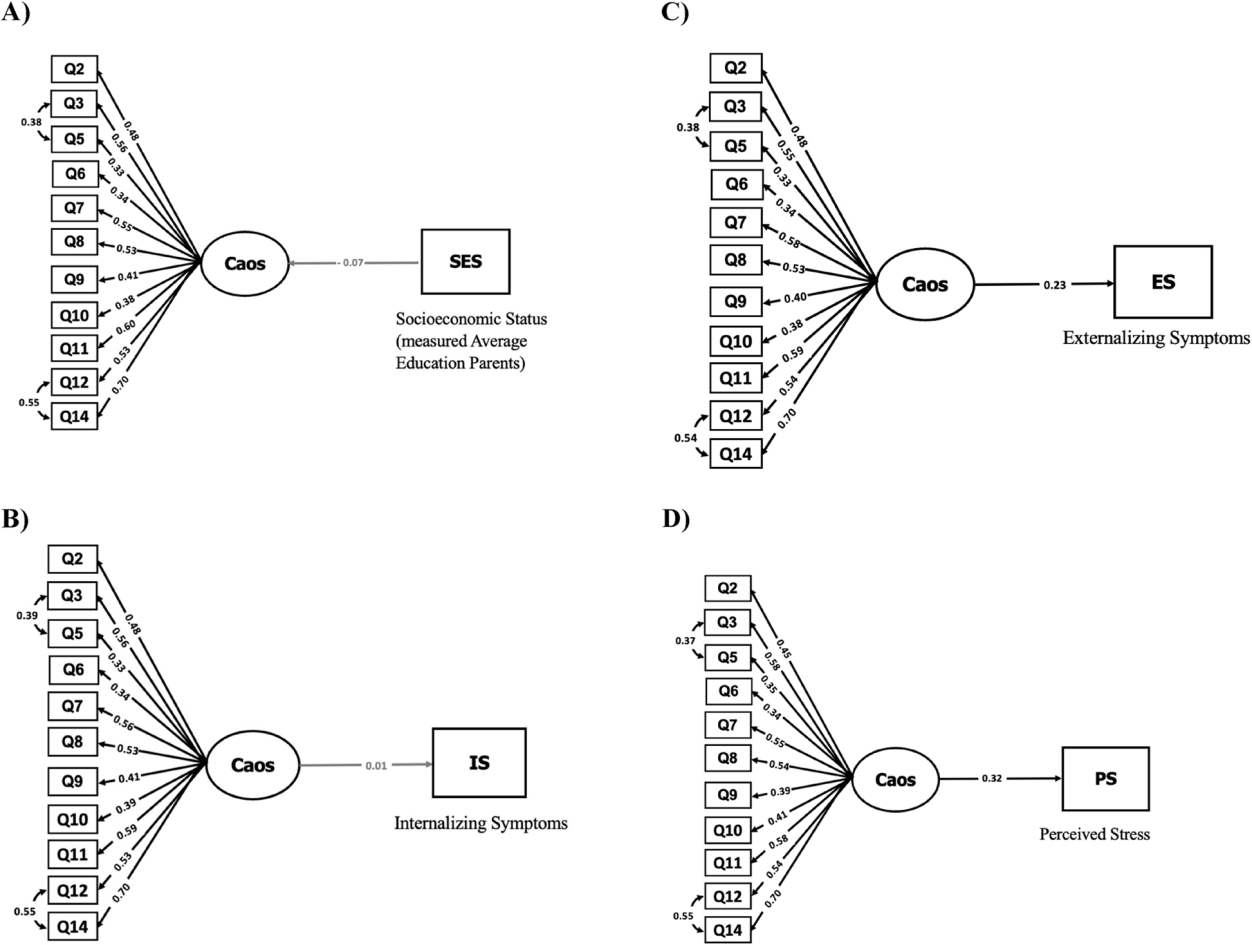
Complete structural equation modeling models of the CAOS scale including associations between the family chaos latent variable and **A** socioeconomic status measured by average years of parental education (SES). **B** internalizing symptoms (IS), **C** externalizing symptoms (ES), **D** perceived stress (PS). Variables (study III; *n* = 239). Note: Q = question/item. Rectangles = indicators/observed variable (scale items); circles = latent variable of chaos; arrows = direction of effects; numbers on arrows pointing to items = factor loadings; number on arrows connecting the latent factor to the other variables = Betas (standardized regression coefficient and effect size); concave arrows: items with correlated errors

Mplus version 8.8 (Muthén & Muthén, 2017) software was used. The CSEM was implemented based on the best-fitting CFA results to investigate the nomological relationship with other variables previously described in the literature as reflecting constructs influenced by and/or related to chaos in the family environment. Each of the following variables was tested in separate models (Fig. 1, which includes factor loadings): (1) family SES, assessed indirectly by the parents’ average years of schooling; (2) symptoms of internalizing and (3) externalizing behaviors assessed by parents; (4) self-report of perceived stress assessed by the adolescents. These nomological networks were used to present evidence of convergent validity (AERA et al. 2014; Preckel & Brunner, 2020). The algorithm used (RDWLS) and the adjustment indices were the same as for the AFC, described above. The direction of the effects in the models varied between variables with respect to possible causal relationships: chaos in the family environment makes more sense as being caused (rather than being the cause) of parents’ education, while the other variables are considered as consequences of family chaos (see the direction of the arrows to the right of the latent variables in Fig. 1).

### Results and discussion

Participants had a mean age of 12.51 (SD = 1.84), ranging from 9 to 16 years (38% were boys). The frequency distribution of participants per age was: 11 9-yer-olds; 25 10-year-olds; 44 11-year-olds; 47 12-year-olds; 28 13-year-olds; 34 14-year-olds; 48 15-year-olds; and 2 16-year-olds. The average years of parental education used as an SES proxy (Farah, 2017; Korous et al., 2018) was 10.82 (SD = 3.04), ranging from 3 to 19 years of education. The mean scores for internalizing and externalizing behaviors were 12.59 (SD = 7.94) and 9.72 (SD = 7.76), respectively. The mean total perceived stress score was 6.51 (SD = 3.14). The mean total raw score of the CAOS scale was 5.58 (SD = 2.82).

### Confirmatory factor analysis

To test the validity of the internal structure of the Brazilian version of the scale, CFA was used to form a latent variable with a unidimensional structure in accordance with the EFA findings in the sample of mothers in study II, as well as the theoretical conceptualization of the proponents of the original CHAOS scale (Matheny et al., 1995). This model presented unsatisfactory fit indices (original model, Tables 2 and 3): the *p* values of χ^2^/ were less than 0.05 and the CFI, TLI, and SRMR indices did not support an adequate fit of the data to the model, although the RMSEA indices and the chi-square ratio per degrees of freedom were acceptable. Items with the lowest factor loadings (items 1, 4, 13, and 15) were then removed to test a respecified model (respecified model 1: Tables 2 and 3). Three of these (items 4, 13, and 15) were also suggested for removal by the MSA index in the factor analysis. Regarding item 13, it is important to consider that the context of telephone use at the time the scale was created in the 1990s is somewhat different from today when telephones are integral parts of people’s lives. This alone does not explain our results, however, because this item already presented low correlations with the sum score in Matheny et al.’s (1995) study, together with item 15, which we also removed here. This confirms that both these items are indeed less associated with the remainder and can bias results if they are included in the CAOS scale. Items 1 and 4, however, were only less associated with the others in the Brazilian version. A possible reason for this is that home commotion (item 1) and running late (item 4) might not be associated with family chaos equivalently across different cultures.

**Table 2:** Fit indices of the tested one-factor confirmatory factor analyses models of the CAOS scale (study III; *N* = 239)

Models	*χ*^2^ (gl)	*χ*^2^/gl	CFI	TLI	SRMR	RMSEA	RMSEA (90% CI)
Original	133.23*(90)	1,48	0.88	0.86	0.10	0.04	(0.02–0.06)
Respecified 1	78.34*(44)	1,78	0.90	0.88	0.09	0.05	(0.03–0.07)
Respecified 2	55.49*(42)	1,32	0.96	0.95	0.08	0.03	(0.00–0.06)

*χ2* chi-square, *gl* degrees of freedom, *CFI* Comparative Fit Index, *TLI* Tucker-Lewis Index, *SRMR* standardized root mean square residual, *RMSEA* root mean square error of approximation, *CI* confidence interval *p < 0.05 of *χ*2. The respecified model 1 was performed after removing the items with the lowest factor loading. The respecified model 2 was equivalent to the respecified model 1 with the inclusion of two correlated errors (items 3–5 and items 12–14). For details and cut-off values of the fit indices, see the text

**Table 3:** Factor loading per CAOS item in the three tested confirmatory factor analyses models (study III; *N* = 239)

**Items**		**Models**	
	**Original**	**Respecified 1**	**Respecified 2**
1	0.24	–	–
2	0.45	0.45	0.48
3	0.57	0.58	0.56
4	0.17	–	–
5	0.37	0.39	0.33
6	0.35	0.32	0.34
7	0.54	0.53	0.56
8	0.49	0.50	0.53
9	0.37	0.39	0.41
10	0.36	0.38	0.39
11	0.54	0.55	0.59
12	0.69	0.68	0.52
13	0.19	–	–
14	0.84	0.82	0.70
15	0.20	–	–

The respecified model 1 was carried out after removing the items with the lowest factor loading (items 1, 4, 13, and 15). The respecified model 2 was equivalent to the latter but also included two correlated errors (items 3–5 and 12–14). For details and fit indices, see the text. Factor loadings below 0.30 are regarded as inadequate (in grey)

After removing these three items with low factor loading, there was a considerable improvement in all adjustment indices but these values were still not acceptable considering several cutoff points. For this reason, correlated errors were added (respecified model 2; Tables 2 and 3) between the pairs of items 3 and 5, and 12 and 14, as they presented an MI > 10. This reflects correlations among indicators that are not explained by the modeled latent factor (family chaos itself). We identified specific associations beyond this between the two sets of items with high MIs that could account for this and justified this procedure: items 3 and 5 are about being in a rush and items 12 and 14 are about relaxing. With these modifications, the fit indices became acceptable Given the results of these analyses, the respecified model 2 was considered in the CSEM.

### Complete structural equation modeling–convergent validity

Figure 1A–D shows the results of the *CSEM* based on the sample of adolescents, with the objective of identifying the relationships of the latent variable of the CAOS scale of the respecified model 2 (Tables 2 and 3) with other variables already described in the literature as related to the home chaotic environment construct. We started with parents' average level of education because, if it were associated with the latent chaos score, it would be important to control for it in the other models because SES has been found to relate to the other tested outcomes. Next, the relationship of the latent variable with internalizing and externalizing symptoms (both coming from the CBCL scale completed by the parents in respect of their children) and perceived stress, reported by adolescents through the PSS-4 scale, was tested. In total, therefore, four models were created, one for each of these four variables/outcomes.

Table 4 shows the adjustment indices for all tested models, all of which had acceptable fit indices. The CHAOS latent variable was not associated with SES, indicating that this factor did not need to be considered in the other models as a control. This lack of association between scores on the CAOS scale and parental education differs from some results found in the literature, which showed a relationship in samples with adolescents and children in samples from developed countries (Brown et al., 2019; Philbrook et al., 2020). This discrepancy can be explained in several ways. First, Brown et al. (2019) and Philbrook et al. (2020) used raw CHAOS scores, and not latent scale scores as done here, which does not correct for measurement errors, or for the different levels of correlation between the item scores on the scale. Another possible explanation is that although parental schooling is a good proxy for SES, it may tap SES differently from the measures used in these other studies (e.g., family income, purchasing power, and accumulated assets, among others) (Farah, 2017; Korous et al., 2018). Moreover, the extent of differences in SES varies greatly from country to country, being much smaller in developed nations than in Brazil, contributing to confusing comparability between publications. Notwithstanding, the fact that there was a wide range of parental education in our sample suggests that this factor was not necessarily responsible for chaos in the family environment. Thus, this scale does not seem particularly sensitive to this variable, at least in families of typically developing adolescents from schools in the city of São Paulo.

**Table 4:** Fit indices of the one-factor complete structural equation models to test for convergent validity of the CAOS scale (*N* = 239)

Associated variables	*χ*^2^ (gl)	*χ*^2^/gl	CFI	TLI	SRMR	RMSEA	RMSEA (90% CI)
Average parental education (years)	66.48*(52)	1,27	0.95	0.94	0.08	0.03	(0.00–0.05)
Internalizing symptoms	72.28*(52)	1,39	0.94	0.92	0.08	0.04	(0.01–0.06)
**Externalizing symptoms**	63.42*(52)	**1,21**	**0.96**	**0.96**	**0.07**	**0.03**	**(0.00–0.05)**
**Perceived stress**	67.02*(52)	**1,28**	**0.96**	**0.94**	**0.08**	**0.03**	**(0.00–0.05)**

*χ*2 chi-square, *gl* degrees of freedom, *CFI* Comparative Fit Index, *TLI* Tucker-Lewis Index, SRMR standardized root mean square residual, *RMSEA* root mean square error of approximation, *CI* confidence interval *p < 0.05 of χ2. Variables in bold had significant Beta (*p* < 0.05). For acceptable values of fit indices, see the text

In contrast, there was a relationship between the latent variable of family chaos and adolescents’ externalizing symptoms (*B* = 0.23, *p* = 0.006, standard error = 0.08), assessed by parents, and perceived stress (*B* = 0.32, *p* = 0.00, standard error = 0.08), assessed by the adolescents themselves which evidenced convergent validity of the Brazilian version of the CHAOS (AERA et al. 2014). This contributes to the construction of a good nomological network: the manifestation of the representation of constructs of interest (in this case, family chaos as a latent measure) in relation to other variables (Preckel & Brunner, 2020). More specifically, the results are in line with the literature regarding externalizing symptoms (Vilsaint et al., 2013; Wang et al., 2012; Wilhoit et al., 2021). With respect to stress, there are also consistent references to its association with family chaos (Ellis & Del Giudice, 2019), particularly physiological stress, which has been measured by changes in cortisol concentrations in samples of children (e.g., Brown et al., 2019; Doom et al., 2018; Marsh et al., 2020), which is line with the perceptions of higher stress found in the current study and the difficulties in dealing with the unpredictability of the family environment.

However, the latent variable of family chaos was less sensitive to internalizing symptoms in the tested adolescents. When considering this result, it should be borne in mind that parents and teenagers perceive behavior differently, and this study only considered parental perceptions. It is therefore possible that parents were better able to identify externalizing behaviors, such as aggression, hyperactivity, and difficulty controlling impulses in their offspring than internalizing symptoms, such as feelings of inferiority, withdrawal, anxiety, and depression (Salbach-Andrae et al., 2009), which adolescents may be less prone to show.

Furthermore, it must be mentioned that the relationships between latent family chaos and externalizing behaviors assessed by parents and stress perceived by adolescents were not very high, but this may be explained by the characteristics of our sample: adolescents with typical development and whose parents agreed to participate in the study. In families such as these, it would not be surprising that problems of this nature would be rarer than in families with adolescents who present clinical and behavioral problems.

## General discussion

In the present study, the translation and cultural adaptation process (Borsa et al., 2012) of the CHAOS demonstrated satisfactory results with respect to content validity regarding the semantic equivalence for the tested Brazilian sample (study I). The EFA examined the internal structure of the scale (study II, in a sample of mothers) and showed that the scale is unifactorial, with the adjustment indices being acceptable, thus confirming its original conception of being a scale that reflects a single construct (Matheny et al., 1995). The CFA confirmed the factor structure (study III in a sample of adolescents), but only after removing items with very low factor loadings (1, 4, 13, and 15), which did not significantly contribute to the measurement of this construct (Howard, 2016), and with the addition of correlated errors between two pairs of items to the respecified model. In study III, the convergent validity of the latent variable with these changes was also determined (Marsh et al., 2020), corroborating the association between chaos in the family environment and externalizing symptoms assessed by parents (Vilsaint et al., 2013; Wang et al., 2012; Wilhoit et al., 2021) and self-rated perceived stress by adolescents (Brown et al., 2019; Doom et al., 2018). Together, these results indicate that the Brazilian version of the scale can be used for certain purposes, but there are some limitations that will be addressed below.

Regarding the results of study III, unlike some publications from other countries with children, rather than adolescents (Vilsaint, et al., 2013; Wang et al., 2012; Wilhoit et al., 2021), and comprising samples with high social vulnerability (Vilsaint et al., 2013; Wilhoit et al., 2021), no evidence was found of the association between chaos in the family environment and internalizing problems, which can be explained by the fact that our sample consisted of adolescents with typical development and/or possibly due to potential difficulty of parents in identifying internalizing symptoms in their adolescent children, which may be biased by cultural beliefs (Korous et al., 2018). Additionally, we failed to find an association between the latent variable of family chaos and the average level of parental education that have been reported by others (Brown et al., 2019; Philbrook et al., 2020), possibly due to the different methods used to assess SES (Farah, 2017; Korous et al., 2018), and the different characteristics of the samples used here and in previous studies that investigated this issue. Our results also show that it is possible to find the expected associations of family chaos with behavioral outcomes in adolescents when the adolescents themselves (and not their parents), fill out the CAOS. This means the scale is easily understood by this age, as long as adolescents have sufficient reading skills (in the present case, they were typically developing and had not been held back at school). Hence, our findings show it is possible to extend the use of the scale to this demographic group.

The present set of studies reported here was pioneering as they employed current psychometric methods using different techniques to investigate various types of validity (construct, configurable structure, and internal and convergent validity) in different samples. Previous validity studies regarding the CHAOS mainly used metrics such as Cronbach's alpha (e.g., Eom et al., 2021; Matheny et al., 1995), which are no longer considered suitable for this purpose (McNeish, 2018; Schweizer, 2011), or used latent factors that did not comply with theory as detailed below (Sánchez-Mondragón & Flores Herrera, 2019; Shervey, 2013).

Our set of studies was also the first in Brazil to present and validate a scale that measures chaos in the family environment, an important construct related to family health and the innermost level of the bioecological model (Bronfenbrenner, 2005; Bronfenbrenner & Morris, 1998). Although other scales that assess family life, such as the Conflict Tactics Scales (CTS), the Family Adaptability and Cohesion Evaluation Scale IV (FACES IV) (Hasselmann & Reichenheim, 2003; Santos et al., 2017), and the Family Environment Scale (FES) are available in Brazil, their focus is mainly on family violence and group cohesion. Moreover, they are generally much longer, take more time to complete, and are not suitable for use with children and/or adolescents. In contrast, the Brazilian CAOS scale is much shorter than the aforementioned ones, can be completed both by parents and adolescents, and is copyright-free, so can be used even by researchers with low funding. Furthermore, even in our sample of adolescents, who were typically developing, it was possible to identify the relationship between family chaos, measured as a latent variable (after removing some items and including correlated errors), and externalizing behaviors assessed by parents, as well as perceived stress reported by adolescents. This reinforces its suitability and shows that the scale was more sensitive to these factors than to an indicator of SES (parental education), suggesting that the scale measures, at a latent level, a construct that may prove to be little influenced by the great social inequality found in Brazil.

Despite the fact that our unidimensional latent CAOS factor was psychometrically adequate and associated with expected outcomes, more studies are needed to determine the suitability of using the total sum score (raw, non-latent), containing responses to all 15 items of the internationally used scale. This is so because although we found a latent variable with acceptable fit indices, this was achieved only after removing four items (which had low factor loadings, meaning they were not associated with the latent factor reflected by the remaining indicators) and including some correlated errors, after which some remaining items still had relatively low factor loading (items 5 and 6, respectively 0.33 and 0.34), although they were above the minimum acceptable level of 0.30 (Field, 2020; Howard, 2016). In other words, not all items of the original scale seem to measure the same construct. Some of them contribute very little to it and can even introduce bias and/or reduce the validity of the scale, which justifies their exclusion, especially as no other study has demonstrated the psychometric adequacy of all the CHAOS items as they were originally conceptualized (with dichotomous answers). We believe that the inadequacy of the excluded items is unlikely to be accounted for by poor translations of the scale into Portuguese in Study I. The reason is that except for one of the two back-translations of item 15, all other translations/back-translations in duplicates of all the four excluded items were regarded as presenting good semantic equivalence and referential meaning. Items 13 and 15 may indeed be weakly related to home chaos because Matheny et al. (1995) had already called attention to the fact they correlated little with the sum score of the scale, which we confirmed here using a more adequate psychometric approach. Differently, the other two items that were removed (items 1 and 4), pertaining to commotion in the home and staying on top of things, respectively, were specific to our sample and may indicate sociocultural aspects that vary among populations in terms of chaotic home environments. In terms of the similarity with other publications regarding our factor models, the only two studies we found that used SEM to analyze the full CHAOS scale either: (1) did not succeed in obtaining an acceptable fitting model (with one, two, and three factors: Shervey, 2013); or (2) found a good model (Sánchez-Mondragón & Flores Herrera, 2019), but not a single factor one as described here; rather, the published model was a three-intercorrelated factor solution which was proposed based on Shervey (2013), whose similar model was far from ideal. Moreover, to reach this good model Sánchez-Mondragón and Flores Herrera (2019) had to exclude 6 of the 15 items, while we removed only four items to form a single factor. In this respect, it seems our data concur with those of Sánchez-Mondragón and Flores Herrera (2019) to a certain degree. Some items of the CHAOS actually do seem to share little variance with others, at least when it comes to the versions in Spanish and Portuguese. It must also be mentioned that our results are not comparable to those of Sánchez-Mondragón and Flores Herrera (2019) for another reason: they employed the Likert scale instead of the true/false responses proposed in the original CHAOS. Because of all these differences between our and their study (version of the CHAOS, model configuration, and type of indicator), it is not possible to determine which items are the least adequate by comparing results. Notwithstanding, both our, Shervey (2013) and Sánchez-Mondragón and Flores Herrera’s (2019) findings indicate that a reduced CHAOS scale, with several of the questions being removed, might be more psychometrically adequate.

Indeed, the advantage of using reduced scales has been considered in the literature. This not only allows the exclusion of items that share little variance with the others but also can resolve the problem of including items in the scale whose residuals covary, corrected for here in the CFA. Different short versions of the original CHAOS scale (short-CHAOS) are available although their precise origin is unclear (Larsen et al., 2022). One of the most cited ones has six items, two of which, however, are not even part of the original scale proposed by Matheny et al. (1995). This short version also does not appear to have acceptable psychometric characteristics (Larsen et al., 2022). Furthermore, the items in these reduced scales that are common to those studied here did not have the highest factor loadings (items 7 and 14). Because we found that most of the 15 items of the original CHAOS do seem to inter-associate enough to form a single latent factor, future studies should determine which of the 15 questions of the scale can be removed to produce a reduced version that stands up to evaluation using up-to-date psychometric analyses. Another possibility is to explore other factor structures, but they should be grounded in theory which is as yet unavailable and/or validated against other associated constructs beyond psychometric analyses.

This study has some limitations which should be mentioned. First, we used convenience samples from the State of São Paulo, and thus may not be representative of the Brazilian population. Second, the adolescents who completed the scale were typically developing and from families that agreed to participate in the study so may have been less vulnerable, both in respect to having a chaotic family environment and to behavioral problems and stress. Our objective, however, was not to provide data on the appropriateness of the CAOS for all Brazilian nationals of all ages, which would have required a different study design, but, rather, to determine some initial psychometric properties of an adapted CHAOS scale in Portuguese. Third, we altered the wording of one item (item 8) across Study II and II. We also did not investigate non-linear effects, as these types of models have not yet been well established in the literature; nor did we evaluate the effects of gender, which is associated with different patterns of behavior and associations with SES (Korous et al., 2018). Larger samples could also have yielded different results. Finally, we did not explore formative models (ours were reflective models), so it cannot be excluded that a suitability latent composite can be obtained from all 15 items of the scale (Bollen, 2011). Therefore, new studies are recommended to confirm our results in samples with more diverse characteristics and from different contexts in Brazil. In spite of this fact, considering that validity is now understood as a degree (AERA et al. 2014), we have provided evidence of the validity of the CAOS scale (validity of the adaptation/translation of the scale; validity of internal structure with factor analysis and confirmatory analysis, as well as convergent validity). All these models provide a unique and pioneering robustness to our study compared to others available in the literature considering that we used advanced and up-to-date statistical methods and metrics which allowed us to show that the CAOS scale can be used in the Brazilian context despite some shortcomings that must be further adjusted and explored.

Lastly, two other issues should be taken into account regarding CHAOS itself, which was developed in the 1990s, meaning it presents some characteristics that can be criticized in today’s scientific scenario. First, as is still common for most questionnaires, some CHAOS items are reversed scored in an attempt to avoid acquiescence bias (e.g., a tendency to agree with statements when in doubt) and disacquiescence (i.e., a tendency to choose responses that state disagreement), both of which can affect scale validity, particularly in young responders (e.g., Primi et al., 2019). Had we altered the scale in this respect we would have changed its original nature, making results incomparable to those of other publications that used the same questionnaire. Hence, future studies should determine to what extent individual differences in acquiescence/disacquiescence affect the CHAOS scale in its current form and/or whether different psychometric properties arise from changing items so that they all indicate higher or lower chaotic home environments. The second point is that some items may be considered rather old-fashioned (e.g., about telephone use; item 13) and/or be associated with chaotic home environments in a socio-culturally sensitive way. Consequently, cross-cultural invariance testing must be explored to investigate this issue.

## Conclusion

The present study presented sufficient evidence of construct, internal, and convergent validity to indicate the use of the Brazilian adaptation of the Confusion, Hubbub, and Order Scale. However, several items on the scale had very low factorial loading, therefore more studies are needed to determine whether the use of the raw sum of the scores of all items of the scale is an adequate score and whether a possible reduced scale will have better reliability/validity.

### Supplementary Information

Supplementary Material 1: Table 1A. Semantic equivalence of back-translations into Portuguese of the Chaos, Hubbub and Order Scale (CHAOS). Table 2A: Brazilian version of the CHAOS scale, titled “Escala de Confusão, Alvoroço e Ordem no Sistema familiar (CAOS)” and the instructions (*instruções*) for filling it out in Portuguese. Table 3A. Covariant matrix of the CAOS items (1-15) in the Exploratory Factor Analysis (Study II; N=180).

## Data Availability

The datasets and software scripts (R, Factor and MPLUS) were available from Open Science Framework in this link: https://osf.io/nrp6z/?view_only=7a7c11fa25ca44fd96c56e98690fa852

## Abbreviations

AERA: American Educational Research Association
CHAOS: Confusion, Hubbub, and Order Scale
CBCL: Child Behavior Checklist
CFA: Confirmatory factor analysis
CFI: Comparative Fit Index
CSEM: Complete Structural Equation Modeling
EFA: Exploratory factor analysis
KMO: Bartlett and Kaiser–Meyer–Olkin
MI: Modification Index
MSA: Measure of sampling adequacy
OSF: Open Science Framework
PSS-4: Perceived Stress Scale
RDWLS: Robust diagonally weighted least squares
RMSEA: Root mean square error of approximation
SD: Standard deviation
TLI: Tucker-Lewis Index
UNESP: Universidade Estadual Paulista “Júlio de Mesquita Filho”
UNIFESP: Federal University of São Paulo
