# Supplementary material for: Adaptation and validation for use in Brazil of the Confusion, Hubbub, and Order Scale (CHAOS)
Source: Psicol Reflex Crit. 2024 Jul 15;37:26. doi: 10.1186/s41155-024-00310-5 (PMC11250710; doi:10.1186/s41155-024-00310-5)
Supplement: Supplementary file 1 — Supplementary Material 1: Table 1A. Semantic equivalence of back-translations into Portuguese of the Chaos, Hubbub and Order Scale (CHAOS). Table 2A: Brazilian version of the CHAOS scale, titled “Escala de Confusão, Alvoroço e Ordem no Sistema familiar (CAOS)” and the instructions (instruções) for filling it out in Portuguese. Table 3A. Covariant matrix of the CAOS items (1-15) in the Exploratory Factor Analysis (Study II; N=180). [file 41155_2024_310_MOESM1_ESM.docx]

**This is an additional (supplementary) file to the paper titled “Adaptation and Validation for Use in Brazil of the Confusion, Hubbub and Order Scale (CHAOS)”.**

**Table 1A. Semantic equivalence of back-translations into Portuguese of the Chaos, Hubbub and Order Scale (CHAOS).**

| **ORIGINAL FORMULATION** | **Translation 1** | **Translation 2** | **Back-translation 1** | **R1 (%)** | **G1** | **Back-translation 2** | **R2 (%)** | | **G2** | **Final version** |
| --- | --- | --- | --- | --- | --- | --- | --- | --- | --- | --- |
| 1.There is very little commotion in our home | Há pouca agitação em nossa casa | Em casa tem pouca agitação | At home there is little commotion | 100 | (U) | There isn’t much unrest in our home | | 90 | (SC) | Há pouca agitação em nossa casa |
| 2.We can usually find things when we need them | Normalmente encontramos as coisas que procuramos | Nós normalmente encontramos as coisas quando precisamos | We normally find things when we need them | 100 | (U) | Normally we find the things we are looking for | | 100 | (U) | Nós normalmente encontramos as coisas quando precisamos |
| 3.We almost always seem to be rushed | Quase sempre estamos com pressa | Nós quase sempre parecemos apressados | We normally appear to be in a hurry | 90 | (SC) | We are almost always in a rush | | 100 | (U) | Quase sempre estamos com pressa |
| 4.We are usually able to stay on top of things | Geralmente somos capazes de estar a par do que está acontecendo | Nós normalmente conseguimos fazer o necessário | We normally manage to get necessary things done | 90 | (SC) | Generally we are able to keep on top of what is happening | | 100 | (U) | Geralmente somos capazes de estar a par do que está acontecendo |
| 5.No matter how hard we try, we always seem to be running late* | Apesar de tentarmos, parece que sempre estamos atrasados | Não importa quanto esforço fazemos estamos sempre atrasados | It does not really matter how much effort we make, we are always late | 90 | (SC) | Although we try it seems as though we are always late | | 100 | (U) | Não importa quanto esforço fazemos, parece que sempre estamos atrasados |
| 6.It’s a real zoo in our home | A nossa casa é uma verdadeira bagunça | Em casa é uma verdadeira bagunça | Home is a real mess | 95 | (U) | Our home is a real mess | | 100 | (U) | A nossa casa é uma verdadeira bagunça |
| 7.At home we can talk to each other without being interrupted | Em casa, nós podemos falar uns com os outros sem sermos interrompidos | Em casa conseguimos conversar um com outro sem que haja interrupções | At home we manage to have conversations without interruptions | 95 | (U) | At home we can talk to each other without being interrupted | | 100 | (U) | Em casa, nós podemos falar uns com os outros sem sermos interrompidos |
| 8.There is often a fuss going on at our home ** | Muitas vezes há uma grande bagunça em casa | Em casa normalmente tem muita confusão | At home there is a lot of commotion | 100 | (U) | There is often a big mess at home | | 100 | (U) | Em casa normalmente tem muita barulheira |
| 9.No matter what our family plans, it usually doesn’t seem to work out | Independentemente do que a nossa família planeje, em geral não dá certo | Não importa o que nossa família planeja normalmente não conseguimos realizar | It does not really matter how well our family plans a head, things never work out in the end | 90 | (SC) | Regardless of what our family plans ahead, it generally doesn’t work out | | 100 | (U) | Independente mente do que a nossa família planeje, em geral não dá certo |
| 10.You can’t hear yourself think in our home | Você não consegue ficar consigo mesmo em nossa casa | Em casa, você não consegue ouvir seu próprio pensamento | It’s impossible to hear yourself think at home | 100 | (U) | You can’t really be yourself in our home | | 90 | (SC) | Em casa, você não consegue ouvir seu próprio pensamento |
| 11.I often get drawn into other people’s arguments at home | Muitas vezes eu me meto em discussões de outras pessoas em casa | Em casa eu normalmente me intrometo nas discussões de outras pessoas | At home I normally allow myself to join in other people’s conversations | 60 | (CC) | I often get involved in other people’s discussions at home | | 100 | (U) | Muitas vezes eu me meto em discussões de outras pessoas em casa |
| 12.Our home is a good place to relax | Nossa casa é um bom lugar para relaxar | Nossa casa é um bom lugar para relaxar | Our home is a great place to relax | 100 | (U) | Our home is a good place to relax | | 100 | (U) | Nossa casa é um bom lugar para relaxar |
| 13.The telephone takes up a lot of our time at home | O telefone ocupa muito do nosso tempo em casa | Em casa gastamos muito tempo no telefone | At home we spend a long time on the phone | 100 | (U) | The phone takes up a lot of our time at home | | 100 | (U) | O telefone ocupa muito do nosso tempo em casa |
| 14.The atmosphere in our home is calm | A atmosfera em nossa casa é calma | O ambiente em casa é calmo | The atmosphere at home is peaceful | 100 | (U) | The atmosphere in our home is calm | | 100 | (U) | O ambiente em casa é calmo |
| 15.First thing in the day, we have a regular routine at home | Acima de tudo, temos uma rotina regular em casa | Em casa, a primeira coisa do dia é ter uma rotina regular | At home, the first thing of the day is to have regular routine | 100 | (U) | Above all else, we have a regular routine at home | | 60 | (CC) | Pela manhã, temos uma rotina regular em casa |

Note: Translations and back-translations 1 and 2 were each carried out by different experts, independently. The semantic equivalence of each translation/backtranslation was determines by a linguist as follows: R1, R2: referential meaning of Retranslations 1 and 2, respectively, on a scale of 0 to 100% (100% being the maximum agreement); G1, G2: general meaning of back-translations 1 and 2, respectively, noted as unchanged (U), slightly changed (SC), considerably changed (CC); extremely changed items were not indicated. Two of the authors selected the most precise translations except that: *A combination of the translations of item 5 was used in the final version; **The translation/backtranslation 2 was prioritized because the other alternative was equivalent to item 6. -Item 8 was later changed to “Em casa tem muitas preocupações desnecessárias” in the study with adolescents to better correspond to the literal meaning according to the suggestion of another expert bilingual judge, who assessed the suitability of the wording for use in young people (see main text for details)-.

**Table 2A: Brazilian version of the CHAOS scale, titled “Escala de Confusão, Alvoroço e Ordem no Sistema familiar (CAOS)” and the instructions (*instruções*) for filling it out in Portuguese.**

Instrução: Leia as frases e faça um “X” na opção que melhor reflete como é na sua casa (verdadeiro ou falso). Se tiver pais separados, considere a casa na qual passa mais tempo.

| **Afirmativas** | **Verdadeiro** | **Falso** |
| --- | --- | --- |
| 1. Há pouca agitação em nossa casa.* |  |  |
| 1. Nós normalmente encontramos as coisas quando precisamos. |  |  |
| 1. Quase sempre estamos com pressa. |  |  |
| 1. Geralmente somos capazes de estar a par (estar sabendo) do que está acontecendo* |  |  |
| 1. Não importa quanto esforço fazemos, parece que sempre estamos atrasados. |  |  |
| 1. A nossa casa é uma verdadeira bagunça. |  |  |
| 1. Em casa, nós podemos falar uns com os outros sem sermos interrompidos. |  |  |
| 1. Em casa tem muitas preocupações desnecessárias. |  |  |
| 1. Independentemente do que a nossa família planeje, em geral não dá certo. |  |  |
| 1. Em casa, você não consegue ouvir seu próprio pensamento. |  |  |
| 1. Muitas vezes eu me meto em discussões de outras pessoas, em casa. |  |  |
| 1. Nossa casa é um bom lugar para relaxar. |  |  |
| 1. O telefone (celular) ocupa muito do nosso tempo em casa. * |  |  |
| 1. O ambiente em casa é calmo. |  |  |
| 1. Pela manhã, temos uma rotina regular em casa. * |  |  |

Note: * Itens that were excluded in the Confirmatory Factor Analysis (see main text).

**Table 3A. Covariant matrix of the CAOS items (1-15) in the Exploratory Factor Analysis (Study II; N=180)**

| ITEMS | 1 | 2 | 3 | 4 | 5 | 6 | 7 | 8 | 9 | 10 | 11 | 12 | 13 | 14 | 15 |
| --- | --- | --- | --- | --- | --- | --- | --- | --- | --- | --- | --- | --- | --- | --- | --- |
| 1 | -- -- |  |  |  |  |  |  |  |  |  |  |  |  |  |  |
| 2 | 0.349 | -- -- | 0.225 | 0.382 | 0.225 | 0.484 | 0.259 | 0.417 | 0.437 | 0.411 | 0.227 | 0.406 | 0.164 | 0.256 | 0.169 |
| 3 | 0.120 | 0.225 | -- -- | 0.021 | 0.689 | 0.313 | 0.291 | 0.363 | 0.118 | 0.092 | 0.183 | 0.221 | -0.084 | 0.477 | 0.029 |
| 4 | 0.253 | 0.382 | 0.021 | -- -- | 0.151 | 0.521 | -0.029 | 0.101 | 0.347 | 0.247 | 0.432 | 0.411 | 0.408 | 0.117 | 0.616 |
| 5 | 0.065 | 0.225 | 0.689 | 0.151 | -- -- | 0.344 | 0.369 | 0.286 | 0.334 | 0.286 | 0.061 | 0.347 | -0.030 | 0.354 | 0.258 |
| 6 | 0.278 | 0.484 | 0.313 | 0.521 | 0.344 | -- -- | 0.199 | 0.321 | 0.355 | 0.212 | 0.494 | 0.411 | 0.209 | 0.327 | 0.379 |
| 7 | 0.541 | 0.259 | 0.291 | -0.029 | 0.369 | 0.199 | -- -- | 0.524 | 0.251 | 0.312 | 0.110 | 0.491 | -0.066 | 0.517 | 0.110 |
| 8 | 0.646 | 0.417 | 0.363 | 0.101 | 0.286 | 0.321 | 0.524 | -- -- | 0.286 | 0.360 | 0.386 | 0.599 | -0.190 | 0.789 | -0.073 |
| 9 | 0.201 | 0.437 | 0.118 | 0.347 | 0.334 | 0.355 | 0.251 | 0.286 | -- -- | 0.570 | 0.555 | 0.413 | -0.117 | 0.117 | 0.162 |
| 10 | 0.232 | 0.411 | 0.092 | 0.247 | 0.286 | 0.212 | 0.312 | 0.360 | 0.570 | -- -- | 0.446 | 0.364 | 0.059 | 0.290 | 0.128 |
| 11 | 0.393 | 0.227 | 0.183 | 0.432 | 0.061 | 0.494 | 0.110 | 0.386 | 0.555 | 0.446 | -- -- | 0.574 | 0.162 | 0.390 | 0.117 |
| 12 | 0.475 | 0.406 | 0.221 | 0.411 | 0.347 | 0.411 | 0.491 | 0.599 | 0.413 | 0.364 | 0.574 | -- -- | -0.015 | 0.684 | 0.326 |
| 13 | -0.097 | 0.164 | -0.084 | 0.408 | -0.030 | 0.209 | -0.066 | -0.190 | -0.117 | 0.059 | 0.162 | -0.015 | -- -- | -0.027 | 0.231 |
| 14 | 0.702 | 0.275 | 0.512 | 0.125 | 0.380 | 0.351 | 0.555 | 0.847 | 0.126 | 0.311 | 0.418 | 0.735 | -0.029 | -- -- | 0.065 |
| 15 | -0.150 | 0.169 | 0.029 | 0.616 | 0.258 | 0.379 | 0.110 | -0.073 | 0.162 | 0.128 | 0.117 | 0.326 | 0.231 | 0.069 | -- -- |
